# Attention to childbirth and delivery in a university hospital: comparison of practices developed after Network Stork *

**DOI:** 10.1590/1518-8345.2643-3139

**Published:** 2019-04-29

**Authors:** Giovanna De Carli Lopes, Annelise de Carvalho Gonçalves, Helga Geremias Gouveia, Cláudia Junqueira Armellini

**Affiliations:** 1 Universidade Federal do Rio Grande do Sul , Escola de Enfermagem , Porto Alegre , RS , Brasil .

**Keywords:** Obstetric Nursing, Obstetric Labor, Midwifery, Hospital Birth Center, Parturition, World Health Organization, Enfermagem Obstétrica, Trabalho de Parto, Assistência ao Parto, Centro Obstétrico, Parto, Organização Mundial da Saúde, Enfermería Obstétrica, Trabajo de Parto, Tocología, Salas de Parto, Parto, Organización Mundial de la Salud

## Abstract

**Objective:**

to compare, after four years of the implementation of the Stork Network, the obstetric practices developed in a university hospital according to the classification of the World Health Organization.

**Method:**

cross-sectional study carried out in the year of adherence to the Stork Network (377 women) and replicated four years later (586 women). Data were obtained through medical records and a structured questionnaire. The Chi-square test was used in the analysis.

**Results:**

four years after the implementation of the Stork Network, in Category A practices (demonstrably useful practices/good practices), there was increased frequency of companions, non-pharmacological methods, skin-to-skin contact and breastfeeding stimulation, and decreased freedom of position/movement. In Category B (harmful practices), there was reduction of trichotomy and increased venoclysis. In Category C (practices with no sufficient evidence), there was increase of Kristeller’s maneuver. In Category D (improperly used practices), the percentage of digital examinations above the recommended level increased, as well as of analgesics and analgesia, and there was decrease of episiotomy.

**Conclusion:**

these findings indicate the maintenance of a technocratic and interventionist assistance and address the need for changes in the obstetric care model. A globally consolidated path is the incorporation of midwife nurses into childbirth for the appropriate use of technologies and the reduction of unnecessary interventions.

## Abstract (pt)

**Objetivo:**

comparar, após transcorridos quatro anos da implementação da Rede Cegonha, as práticas obstétricas desenvolvidas em um hospital universitário segundo classificação da Organização Mundial da Saúde.

**Método:**

estudo transversal realizado no ano de adesão à Rede Cegonha (377 mulheres) e replicado quatro anos após (586 mulheres). Dados obtidos mediante prontuário e questionário estruturado. Na análise, utilizou-se o Teste Qui-quadrado.

**Resultados:**

quatro anos após a Rede Cegonha, dentre as práticas da Categoria A (práticas comprovadamente úteis/boas práticas), aumentou a frequência de acompanhante, de métodos não farmacológicos, de contato pele a pele e de estímulo à amamentação e diminuiu a liberdade de posição/movimentação. Na Categoria B (práticas prejudiciais), houve redução de tricotomia e aumento de venóclise. Na Categoria C (práticas sem evidências suficientes), o Kristeller apresentou aumento. Na Categoria D (práticas utilizadas de modo inadequado), aumentou o percentual de toque vaginal acima do recomendado, de analgésicos e de analgesia e diminuiu a episiotomia.

**Conclusão:**

esses resultados indicam a manutenção de uma assistência tecnocrática e intervencionista e direcionam para a necessidade de mudanças no modelo de atenção obstétrica. Um caminho consolidado mundialmente é a incorporação de enfermeiras obstetras/obstetrizes na assistência ao parto pelo potencial de utilização apropriada de tecnologias e redução de intervenções desnecessárias.

## Abstract (es)

**Objetivo:**

comparar, después de transcurridos cuatro años de la implementación de la Red Cigüeña, las prácticas obstétricas desarrolladas en un hospital universitario según clasificación de la Organización Mundial de la Salud.

**Método:**

estudio transversal realizado en el año de adhesión a la Red Cigüeña (377 mujeres) y replicado cuatro años después (586 mujeres). Datos obtenidos mediante prontuario y cuestionario estructurado. En el análisis, se utilizó el Test Chi-cuadrado.

**Resultados:**

cuatro años después de la Red Cigüeña, entre las prácticas de la Categoría A (prácticas demostradamente útiles/buenas prácticas), aumentó la frecuencia de acompañante, de métodos no farmacológicos, de contacto piel a piel y de estímulo a la lactancia y disminuyó la libertad de posición/movimiento. En la Categoría B (prácticas perjudiciales), hubo reducción de tricotomía y aumento de venoclisis. En la Categoría C (prácticas sin evidencias suficientes), el Kristeller presentó aumento. En la Categoría D (prácticas utilizadas de modo inadecuado), aumentó el porcentaje de toque vaginal más de lo recomendado, de analgésicos y de analgesia y disminuyó la episiotomía.

**Conclusión:**

esos hallazgos indican el mantenimiento de una asistencia tecnocrática e intervencionista y dirigida para la necesidad de cambios en el modelo de atención obstétrica. Un camino consolidado mundialmente es la incorporación de enfermeras obstetras/parteras en la asistencia al parto por el potencial de utilización apropiada de tecnologías y reducción de intervenciones desnecesarias.

## Introduction

In 1996, the World Health Organization (WHO) developed a manual on normal birth ^(^ ^1^ ^)^ aiming at systematizing obstetric practices and making recommendations based on the best available evidence. Some practices, implemented from the institutionalization of childbirth, are still being carried out today, even with little or no scientific evidence to support them ^(^ ^2^ ^)^ .

In Brazil, several strategies have been developed over the past 30 years to improve the quality of care and reduce rates of cesarean section and maternal and neonatal mortality. Some advances have occurred, but morbidity and mortality have not decreased as expected and are still a challenge ^(^ ^3^ ^-^ ^4^ ^)^ .

Childbirth care prevalent today in Brazil is marked by excessive use of hard technologies and medicalization, leading to unnecessary interventions and high cesarean section rates ^(^ ^5^ ^)^ . In addition, almost all deliveries are performed in hospitals (98.4%) and are attended predominantly by obstetricians (88.7%) ^(^ ^6^ ^)^ . This model of childbirth care - centered in the physician and in the hospital care - is characterized as traditional, being the prevalent model in Brazil ^(^ ^7^ ^)^ . It can also be called obstetrician-led model of care, since it is the doctor who determines the care, and the other professionals only have a supporting role ^(^ ^8^ ^)^ . In addition to this, there are two other models: the shared care, in which responsibility for the organization of women’s care from the prenatal to the puerperal period is shared among different professionals; and the midwife-led care, in which these professionals are the main providers of care for women with a regular-risk gestation, whether in primary or tertiary care. When necessary, the woman is referred to the obstetrician or other specialist ^(^ ^8^ ^)^ .

The model of childbirth care adopted by each health institution determines the care practices developed, which consequently affect maternal and neonatal outcomes. Therefore, it is essential to monitor these practices in order to adjust or change the qualification of maternal and neonatal care, since the indicators aimed at this population have shown to be below the expected, considering the predominant obstetric model in Brazil.

Based on this reality and on the need to qualify and organize the care network in the pregnancy-puerperal period, the Brazilian Ministry of Health (MoH) instituted in 2011, within the scope of the Unified Health System (SUS), the Stork Network strategy, which is organized based on four components. One of the actions of the Childbirth and Birth Component refers to the incorporation of health care practices based on scientific evidence, according to the WHO manual ^(^ ^1^ ^)^ .

Several studies have evaluated care practices for childbirth and delivery before ^(^ ^9^ ^-^ ^11^ ^)^ , during ^(^ ^5^ ^,^ ^12^ ^-^ ^13^ ^)^ and after ^(^ ^14^ ^-^ ^16^ ^)^ the implementation of the Stork Network (SN). However, no study was found comparing, at different periods, the care practices carried out in the same maternity hospital after the implementation of the SN in order to analyze the follow-up of these practices and the repercussions related to the qualification of care.

Thus, this study aims to compare the care practices for childbirth and delivery developed in a university hospital in 2012, the year it joined the Stork Network, with those developed in 2016, four years later, according to evidence-based practices recommended by WHO.

## Method

This is a cross-sectional study that included data from two studies conducted at different periods at the Hospital de Clínicas de Porto Alegre (HCPA) - a university hospital in the city of Porto Alegre, Rio Grande do Sul, Brazil, certified by the Baby-Friendly Hospital Initiative (BFHI). The first study was conducted in 2012 ^(^ ^17^ ^)^ , year of implementation of the SN in the maternity ward, and the second was in 2016, four years after it joined the strategy. This maternity ward is a reference for high risk pregnancy, attends mostly through SUS and the model of care is the traditional one ^(^ ^7^ ^)^ .

In order to calculate the sample size of the 2012 survey, the 3,510 deliveries occurred in the year 2010 were considered. There were no data in the literature on the adequacy levels of the humanized care practices, so the sample size was calculated based on 50% adequacy of each practice, 95% confidence interval and 5% margin of error. Thus, the sample consisted of 385 puerperal women. For the 2016 study, considering a power of 80%, significance level of 5%, proportion of breastfeeding in the first hour of 68% (institutional data) and difference between the proportions of the outcomes of the newborn with OR of 0.6 ^(^ ^7^ ^)^ , we reached the sample size of 586 women. For this calculation, in both surveys, the WinPepi program was used.

Some inclusion and exclusion criteria differed between the two surveys. Exclusions were made in the 2012 survey sample to allow comparability of the variables and to respond to the objective of this study, without affecting its power. Thus, the final sample of the 2012 survey was 377 women and the 2016 survey was maintained, with 586 participants.

The present study included women who had given birth at the institution surveyed and whose newborns’ gestational age was ≥ 37 weeks calculated by the Capurro Method. Women who had undergone elective caesarean section with less than two hours of labor and who were hospitalized through a private health care provider (covenant) or by their own cost, as well as cases of twinning, death and fetal malformation were excluded.

Data collection of the 2012 survey was from August to November, and of the 2016 survey was between February and September. Data from the two surveys were obtained through an electronic medical record supplemented with a physical record and a structured questionnaire applied to women. The interview was performed after 12 hours postpartum. The sampling for both surveys was of the consecutive type, that is, all the puerperal women that they met the inclusion criteria were consecutively included according to the order of delivery.

The data collection instruments of the surveys present some differences between them, since for the 2016 survey the instrument was reviewed and improved. For the accomplishment of the present study, a table was elaborated with the variables of both researches in order to compare them. Thus, sociodemographic and obstetric variables and those related to the practices of care for childbirth and delivery were considered for this study, according to the categories proposed by the WHO. They are: Category A - demonstrably useful practices that should be encouraged - also called good practices; Category B - clearly harmful practices that should be eliminated; Category C - Practices that do not have sufficient evidence to support a precise recommendation and should be used with caution until further research clarifies the issue; and Category D - Practices frequently used inappropriately.

The categorical variables were expressed in frequencies and percentages and the comparison between the years 2012 and 2016 was performed through the Chi-square test. Differences were considered significant when the level of significance ( *p* ) was lower than 0.05. The analyzes were performed using SPSS software version 18.

Both studies were approved by the Research Ethics Committee of the institution, under the numbers 120150 and 150591. The terms of the National Health Council were fulfilled through Resolutions 196/96 and 466/12 for the studies of 2012 and 2016, respectively. All the women who agreed to participate in the studies signed an Informed Consent Form.

## Results

The comparison of the sociodemographic and obstetric variables of the participants of the surveys of 2012 and 2016 presented statistically significant differences for self-reported race/color and schooling ( Table 1 ).

**Table 1:** Distribution of absolute (n) and relative (%) frequency of women according to sociodemographic and obstetric aspects and comparison by year. HCPA*, Porto Alegre, RS, Brazil, 2012 and 2016

Variable	2012		2016		p-value ^‡^

	n ^†^	(%)	n ^†^	(%)	
Age					0.382
≤ 20	108	(28.7)	147	(25.1)	
21 to 34 years	238	(63.1)	381	(65.0)	
≥35	31	(8.2)	58	(9.9)	
Self-reported race/color					**<0.001**
Black	55	(14.7)	114	(19.5)	
Brown	104	(27.7)	102	(17.4)	
White	207	(55.2)	364	(62.1)	
Other	9	(2.4)	6	(1.0)	
Schooling					**0.007**
Elementary School	158	(41.9)	187	(31.9)	
High school	189	(50.1)	347	(59.2)	
Higher education	30	(8.0)	52	(8.9)	
Marital status					0.680
Has a partner	338	(90.1)	522	(89.1)	
Does not have a partner	37	(9.9)	64	(10.9)	
Parity					0.090
Nulliparous	197	(52.4)	346	(59.0)	
1 previous delivey	101	(26.9)	145	(24.8)	
≥ 2 previous deliveries	78	(20.7)	95	(16.2)	
Route of birth					0.482
Vaginal	297	(78.8)	449	(76.6)	
Cesarian section	80	(21.2)	137	(23.4)	

*HCPA: Hospital de Clínicas de Porto Alegre; †Considered only valid data; ‡Obtained by Chi-square test

Considering Category A practices, it was verified that, of the eight practices analyzed, five showed a statistically significant difference between the years. Four of them showed an increase in the percentage in 2016: presence pf a companion during the delivery or cesarean section (from 91.0% to 95.7%); use of non-pharmacological methods of pain relief during labor (from 67.9% to 74.2%); skin-to-skin contact (from 14.9% to 60.1%); encouragement by the professional for the mother to breastfeed soon after birth (from 22.1% to 45.0%); and one of them presented a decrease: the freedom of position and movement during labor was from 53.9% to 44.9% in 2016 ( Table 2 ).

**Table 2:** Comparison by year of Category A - Practices demonstrably useful and to be encouraged. HCPA*, Porto Alegre, RS, Brazil, 2012 and 2016

Variable	2012		2016		p-value ^‡^

	n ^†^	(%)	n ^†^	(%)	
Supply of liquids during labor ^§^					
Yes	243	(64.6)	408	(69.6)	0.122
No	133	(35.4)	178	(30.4)	
Companion during labor ^§^					
Yes	366	(97.1)	568	(96.9)	1.000
No	11	(2.9)	18	(3.1)	
Companion during delivery or cesarean section					
Yes	343	(91.0)	561	(95.7)	**0.004**
No	34	(9.0)	25	(4.3)	
Use of NPM ^||^ for pain relief during labor ^§^					
Yes	256	(67.9)	435	(74.2)	**0.040**
No	121	(32.1)	151	(25.8)	
Freedom of position and movement during labor ^§^					
Yes	202	(53.9)	263	(44.9)	**0.008**
No	173	(46.1)	323	(55.1)	
Encouragement to adopt a preferred position in the expulsive period					
Yes	9	(3.1)	28	(6.2)	0.076
No	285	(96.9)	421	(93.8)	
Skin-to-skin contact right after birth					
Yes	56	(14.9)	350	(60.1)	**<0.001**
No	319	(85.1)	232	(39.9)	
Encouragement to breastfeeding soon after birth ^¶^					
Yes	63	(22.1)	200	(45.0)	**<0.001**
No	222	(77.9)	244	(55.0)	

*HCPA: Hospital de Clínicas de Porto Alegre; †Considered only valid data; ‡Obtained by Chi-square test; ||NPM: non-pharmacological methods of pain relief; ¶Only for babies who went to the mother’s lap

Among Category B practices, three variables presented a statistically significant difference. There was a reduction in the percentage of trichotomy for delivery (from 81.3% to 64.0%) and trichotomy performed at the hospital (from 25.3% to 8.8%), as well as an increase in venoclysis during labor (from 85.4% to 97.8%) ( Table 3 ).

**Table 3:** - Comparison by year of Category B - Practices that are clearly harmful or ineffective and that should be eliminated. HCPA*, Porto Alegre, RS, Brazil, 2012 and 2016

Variable	2012		2016		p-value ^‡^

	n ^†^	(%)	n ^†^	(%)	
Enema or other laxative method					
Yes	5	(1.3)	3	(0.5)	0.274
No	371	(98.7)	583	(99.5)	
Place of performance of enema or laxative method ^§^					
At home	1	(25.0)	1	(33.3)	1.000
At the hospital	3	(75.0)	2	(66.7)	
Trichotomy					
Yes	305	(81.3)	375	(64.0)	**<0.001**
No	70	(18.7)	211	(36.0)	
Place of performance of the tricotomy ^§^					
At home	227	(74.7)	341	(91.2)	**<0.001**
At the hospital	77	(25.3)	33	(8.8)	
Venoclysis during labor ^||^					
Yes	322	(85.4)	573	(97.8)	**<0.001**
No	55	(14.6)	13	(2.2)	
Lithotomy position in the expulsive period					
Yes	294	(99.3)	445	(98.7)	0.489
No	2	(0.7)	6	(1.3)	

*HCPA: Hospital de Clínicas de Porto Alegre; †Considered only valid data; ‡Obtained by Chi-square test; §Only those who have been submitted

Regarding the practices investigated in Category C, only the Kristeller’s maneuver had increased percentage in 2016, with a statistically significant difference, from 8.5% to 13.6% ( Table 4 ).

**Table 4:** Comparison by year of Category C - Practices that do not have sufficient evidence to support a clear recommendation and that should be used with caution. HCPA*, Porto Alegre, RS, Brazil, 2012 and 2016

Variable	2012		2016		p-value ^‡^

	n ^†^	(%)	n ^†^	(%)	
Amniotomy during labor					
Yes	224	(81.8)	349	(83.9)	0.529
No	50	(18.2)	67	(16.1)	
Kristeller’s maneuver ^§^					
Yes	25	(8.5)	61	(13.6)	**0.047**
No	268	(91.5)	388	(86.4)	

*HCPA: Hospital de Clínicas de Porto Alegre; †Considered only valid data; ‡Obtained by Chi-square test; §For vaginal delivery only

The results related to Category D identified five variables with a statistically significant difference. In the comparison between the years, the percentage of pharmacological pain relief with analgesics during labor (from 44.4% to 75.1%) and pharmacological relief of pain with analgesia during labor increased (from 20.3% to 45.9%). The variables up to five examinations of digital examinations (from 73.7% to 62.1%) and episiotomy (from 63.6% to 55.0%) presented a percentage reduction. The number of digital examinations above the recommended had a borderline significance (p = 0.055, from 69.5% to 76.8%) ( Table 5 ).

**Table 5:** Comparison by year of Category D - Practices frequently used inappropriately. HCPA*, Porto Alegre, RS, Brazil, 2012 and 2016

Variable	2012		2016		p-value ^‡^

	n ^†^	(%)	n ^†^	(%)	
Pharmacological relief of pain with analgesics during labor					**<0.001**
Yes	143	(44.4)	438	(75.1)	
No	179	(55.6)	145	(24.9)	
Pharmacological relief of pain with analgesia during labor					**<0.001**
Yes	76	(20.3)	269	(45.9)	
No	298	(79.7)	317	(54.1)	
Use of oxytocin					0.568
Yes	258	(80.1)	480	(81.9)	
No	64	(19.9)	106	(18.1)	
Number of digital examinations performed ^§^					**0.008**
Up to 5	165	(73.7)	323	(62.1)	
6 to 10	55	(24.5)	177	(34.0)	
11 or more	4	(1.8)	20	(3.9)	
Adequacy of the number of examinations according to the Ministry of Health ^||^					**0.055**
Below recommended	25	(11.2)	53	(10.5)	
As recommended	43	(19.3)	64	(12.7)	
Above recommended	155	(69.5)	388	(76.8)	
Woman’s assessment on the number of examinations					0.120
Too little	23	(6.1)	22	(3.8)	
Too much	61	(16.2)	115	(19.7)	
Adequate	292	(77.7)	446	(76.5)	
Transfer of the woman to another room at the beginning of the expulsion period					0.145
Yes	276	(93.6)	397	(90.2)	
No	19	(6.4)	43	(9.8)	
Delivery with forceps					0.636
Yes	14	(4.7)	26	(5.8)	
No	283	(95.3)	423	(94.2)	
Episiotomy					**0.024**
Yes	189	(63.6)	247	(55.0)	
No	108	(36.4)	202	(45.0)	
Anesthesia before episiotomy					0.699
Yes	142	(93.4)	223	(94.9)	
No	10	(6.6)	12	(5.1)	

*HCPA: Hospital de Clínicas de Porto Alegre; †Considered only valid data; ‡Obtained by Chi-square test; §Not included women who answered “I do not remember”; ||Only for the valid data of the variables “hospitalization time” and “number of examinations”

## Discussion

The obstetric practices with statistical significance analyzed in this study and belonging to Categories A, B, C and D proposed by WHO ^(^ ^1^ ^)^ did not present the same behavior in relation to the expectations of improvement of their percentages after four years of the implementation of SN in the institution.

Regarding the good practices (Category A) evaluated in this research, there was highlight to the high proportion of companions during the delivery/cesarean section, both in 2012 (91.0%) and in 2016 (96.9%), increasing 5.2% four years after the implementation of the Stork Network. However, that the presence of a companion is provided by law in Brazil since 2005 ^(^ ^18^ ^)^ . A national study that evaluated the presence of companions in public and private hospitals found low proportions thereof during delivery (32.7%) and found that women with deliveries assisted by midwife nurses had a higher percentage of presence of companions at all times (27.2%) when compared to those who were attended by physicians (15.1%) ^(^ ^19^ ^)^ . Rates of presence of companions above 90% in Brazilian institutions, similar to those found in the present study, were found only in Normal Delivery Centers (NDC) ^(^ ^10^ ^,^ ^20^ ^)^ . A Cochrane’s systematic review recommends that continued support be provided by a trained professional that does not make part of the woman’s social circle or of the institution’s care team, as the results have proven to be most effective. However, being monitored by someone who is not trained, such as the partner, is still better than having no companion ^(^ ^21^ ^)^ .

Another practice that improves the experience with the birth is the use of NPM for pain relief, by the decreased need of pharmacological resources ^(^ ^1^ ^)^ , making labor less invasive and less stressful. Similar result was found in a systematic review, in which, in addition to relieving pain, NPM also improved the experience with childbirth when compared with placebo or standard treatment. In addition, some methods are associated with decreased need for forceps/vacuum and cesarean section ^(^ ^22^ ^)^ .

In this study, although the proportion of NPM use has increased by 9.3%, with a rate of 74.2% in 2016, higher rates were found in the literature for both the collaborative model (85.0%) and the traditional model of care (78.9%) ^(^ ^7^ ^)^ . Even though in Brazil, regardless of gestational risk, low rates of NPM use were identified ^(^ ^5^ ^)^ . Other studies have evidenced the universal use of NPM in deliveries attended by midwife nurses both in hospital ^(^ ^23^ ^)^ and in NDCs ^(^ ^10^ ^)^ .

With regard to the freedom of movement and position of the parturient, there was a reduction of this practice in 16.7% over the four years of the SN. The rate found in 2016 was 44.9%, a result lower than the average in the South region (56.3%), as evidenced by the survey Being Born in Brazil ^(^ ^5^ ^)^ . The rates of this practice in hospitals were low before the implementation of SN, regardless of the professional who attended the delivery ^(^ ^24^ ^-^ ^25^ ^)^ . In the NDCs, this rate was higher ^(^ ^10^ ^,^ ^26^ ^)^ , even before the SN. This scenario has partially modified after the NS, since there was an increase in the freedom of position and movement in the deliveries attended by midwife nurses ^(^ ^20^ ^)^ and in hospitals with a collaborative model ^(^ ^13^ ^,^ ^27^ ^)^ .

The freedom of movement and of position during labor is of great clinical importance because the supine position during this period affects the blood flow to the uterus, reducing the blood flow reaching the fetus due to the weight of the uterus that compresses the vena cava when woman is laid down, thus compromising the fetal condition. In addition, the supine position may also reduce the intensity of contractions, thus interfering with the progression of labor. Adopting non-supine positions may make the labor less painful, as there is less need for analgesia and correction of the dynamics with oxytocin ^(^ ^1^ ^)^ . Systematic review by Cochrane concluded that the practice of wandering and remaining in vertical positions is associated with shortening of the labor and the lower probability of cesarean section and analgesia ^(^ ^28^ ^)^ .

The low prevalence of freedom of position and movement found in this study can be explained by the increase in venoclysis, since venous hydration makes it difficult to change position and ambulation of women ^(^ ^1^ ^)^ . Many maternity hospitals do not have space for ambulation, which does not correspond to the reality of the maternity ward studied, which has seven individual pre-delivery rooms, with possibility of moving.

As for skin-to-skin contact, the WHO ^(^ ^1^ ^)^ does not determine a minimum time for this practice, but the BFHI ^(^ ^29^ ^)^ recommends that it should occur immediately at birth or within five minutes and last at least one hour. Studies performed in hospitals certified by the BFHI presented better results on skin-to-skin contact when compared to those non-certified ^(^ ^12^ ^,^ ^30^ ^-^ ^31^ ^)^ . In this research, whose maternity ward is accredited by the BFHI, the percentage of this practice in 2016 was 60.1%, showing an increase of 303.3%. This finding is higher than the average of the South region (32.5%), of Brazilian capitals (35.0%) and of hospitals certified by the BFHI (38.1%), according to a national survey ^(^ ^12^ ^)^ . However, it also presented a proportion below that found by another public Baby-Friendly hospital(72.4%) ^(^ ^32^ ^)^ . According to a Cochrane’s systematic review, early skin-to-skin contact promotes cardiorespiratory stability (better fetal heart rate, respiratory rate, and oxygen saturation parameters) and increases newborns’ blood glucose levels ^(^ ^33^ ^)^ .

Another effective practice of newborn care is the encouragement of breastfeeding soon after birth. The WHO recommends that this encouragement should start within the first hour after delivery ^(^ ^1^ ^)^ . According to a systematic review, breastfeeding in the first hour of life is associated with increased breastfeeding effectiveness and duration of breastfeeding ^(^ ^33^ ^)^ , in addition to being associated with the reduction of neonatal mortality, especially in developing countries ^(^ ^34^ ^)^ .

This practice increased by 103.6% in the delivery/cesarean section room. The percentage of 45.0% obtained in this research in 2016 was above the national average (16.1%), the South region (22.5%), the capitals (20.1%) and the Baby-Friendly hospitals (24.0%), according to a Brazilian survey ^(^ ^12^ ^)^ . Even before the SN, BFHI-certified institutions already had better breastfeeding rates in the first hour of life ^(^ ^21^ ^)^ . The hospital in this study has been certified by the BFHI since 1997. Thus, encouraging breastfeeding in the first hour of life remains a challenge and requires breakthroughs.

Among Category B practices (harmful practices), trichotomy decreased by 21.3%, and was performed in 8.8% of women in 2016. When the place of performance was analyzed, it was verified that there was an increase in trichotomies prior to hospitalization and consequent reduction of this in-hospital practice.

As in this research, other studies also found a reduction in the practice of trichotomy after the SN ^(^ ^35^ ^-^ ^36^ ^)^ . Even so, this harmful practice has been maintained. Opposing this scenario, three public institutions have demonstrated that it is feasible to abolish trichotomy even if the episiotomy is practiced, without interfering in the quality of care ^(^ ^10^ ^,^ ^13^ ^)^ . Trichotomy was incorporated into the obstetric routine under the guise of reducing infections and facilitating the suturing of episiotomy. However, the risk for infection is not reduced; on the contrary, trichotomy may increase the risk of HIV and hepatitis, both for the professional and for the woman, becoming an unnecessary procedure, which should not be performed, unless the woman requests ^(^ ^1^ ^)^ . A systematic review compared trichotomy with the practice of cutting the hair if necessary and did not find differences for several outcomes, including perineal injury infection (either by laceration or episiotomy), and also indicated adverse effects of trichotomy, such as irritation, redness, burning and itching. Thus, the authors concluded that there is insufficient evidence to recommend trichotomy at admission to labor ^(^ ^37^ ^)^ .

Another harmful practice that also stood out was the venoclysis, with an increase of 14.5%, being used routinely and almost universally four years after the SN. In other studies, there were high rates of venoclysis (73.6%) before SN ^(^ ^24^ ^)^ . After its implementation, there was a reduction ^(^ ^38^ ^)^ and, in some cases, it was banished of routine practice in hospitals ^(^ ^39^ ^)^ and in NDCs ^(^ ^10^ ^)^ . A national survey ^(^ ^5^ ^)^ identified a 73.8% rate of venoclysis for women at normal risk and 76.7% for high risk women. The region of the country that most practiced this intervention was the Southeast one (76.0%) and, in third place, the South region (72.9%). These results were lower than those presented by this survey in 2012 (85.4%) and in 2016 (97.8%).

The prophylactic insertion of a venous catheter under the guise of the possible need thereof is not justified because, in addition to generating more costs, it hinders the free change of position and movement of the woman ^(^ ^1^ ^)^ and facilitates other unnecessary interventions. In addition, it is recommended that the replacement of the energy expended by the woman be performed by oral intake of liquids and light meals, and not by intravenous infusion of fluids ^(^ ^1^ ^)^ . A systematic review of Cochrane concluded that there is no robust evidence to recommend routine intravenous fluids administration during labor ^(^ ^40^ ^)^ .

Regarding Category C practices, the Kristeller’s maneuver, although initially classified by the WHO as a practice that does not have sufficient evidence to support a precise recommendation and that should be used with caution until further research clarifies the issue ^(^ ^1^ ^)^ , is currently a practice based on high-level investigations. According to a systematic review, the current evidence is insufficient to support Kristeller’s routine use, either by hand or by wearing a belt or any other method, since perineal lesion was increased with both techniques ^(^ ^41^ ^)^ . In addition, Kristeller is currently understood as obstetric violence ^(^ ^27^ ^)^ , since it is characterized as an unnecessary and harmful procedure, which can lead to physical and psychological trauma.

Even so, in this research, the maintenance of this practice was evidenced, with a rate of 13.6% in 2016, showing an increase of 60.0% four years after the SN. Other studies showed a tendency to decrease Kristeller after the SN, with rates of 55.4% ^(^ ^35^ ^)^ before the strategy and 9.0% ^(^ ^27^ ^)^ after it. The survey is Being Born in Brazil ^(^ ^5^ ^)^ pointed out that Kristeller was performed in 37.3% of the women at normal risk and 33.9% in those at high risk, indicating that this procedure is not related to maternal or fetal conditions. In addition, a recent study has shown that obstetricians perform more Kristeller (38.7%) than midwife nurses (27.2%) ^(^ ^42^ ^)^ .

With regard to Category D practices (frequently inappropriately used practices), although there was an increase of 69.1% and 126.0% in the proportion of women who received analgesics and analgesia, respectively, there is no minimum or appropriate rate for these practices in the literature ^(^ ^1^ ^,^ ^43^ ^)^ . There are few Brazilian studies that have evaluated the proportion of analgesics use during labor, ranging from 4.1% ^(^ ^44^ ^)^ to 97.1% ^(^ ^9^ ^)^ in hospitals and 22.4% ^(^ ^9^ ^)^ in NDCs. A systematic review of the Cochrane library on non-opioid pain management drugs concluded that the evidence is insufficient to support its use as an isolated method for pain relief in labor ^(^ ^43^ ^)^ .

The increases use of intravenous analgesics (from 44.4% to 75.1%) found in this study can be explained by the almost universal performance of venous puncture, which facilitates the practice of this intervention. Another possible explanation would be the reduction of freedom of position and movement, which may increase pain and, consequently, the request for pharmacological resources. In this way, unnecessary practices end up leading to other unnecessary practices.

The analgesia rate found in this study was 45.9% in 2016. Studies showed low rates of practice before the SN (7.7%) ^(^ ^35^ ^)^ and increase after (16.0%) ^(^ ^38^ ^)^ . The survey is Being Born in Brazil ^(^ ^5^ ^)^ found for Brazilian women a lower proportion of analgesia than this study, that is, 33.9%. Studies that evaluated different models of care to delivery found higher frequencies of analgesia in maternity hospitals with a collaborative model when compared to maternity hospitals with a traditional model of care ^(^ ^7^ ^,^ ^45^ ^)^ . Although the procedure represents the medicalization of childbirth, the selective and restricted offer of analgesia is contrary to the philosophy of humanization at birth ^(^ ^45^ ^)^ . However, it is known that the demand for analgesia may be lower in midwife-led models of care ^(^ ^8^ ^)^ . The demand for this procedure is multifactorial and culturally dependent, but the NPMs may be undervalued or used inappropriately, since, even with an increase in their use, the demand for analgesia has also increased. Non-invasive practices for pain relief also require technical knowledge by professionals and should be the first choice. A study evaluating 27 systematic reviews of the Cochrane Library found that there is sufficient evidence in the literature to associate analgesia with cesarean section ^(^ ^46^ ^)^ .

Another procedure that is also not risk free is the digital examination, since its improper practice may result in maternal and neonatal infection ^(^ ^1^ ^)^ . Therefore, the number of examinations should be limited to what is strictly necessary, that is, during the dilation phase, a digital examination every four hours is enough ^(^ ^1^ ^,^ ^47^ ^)^ . A systematic review comparing this practice performed every two hours with that performed every four hours reported that no differences were found in duration of labor, correction of dynamics with oxytocin, use of epidural analgesia and rates of cesarean section, spontaneous vaginal delivery and operative vaginal delivery ^(^ ^48^ ^)^ .

Although the number of digital examinations has increased by 111.0% for the category “11 examinations or more”, the variable “number of digital examinations performed” alone has little clinical relevance, since the frequency of this practice should be determined by the labor time. Thus, the variable “adequacy of the number of examinations” was created, which relates the number of examinations performed (except that of hospital admission) with the length of stay until delivery, according to the MoH and WHO recommendations. Even after this adjustment, the proportion of women who had undergone digital examinations above the recommended level was 76.8%, with an increase of 10.5% (borderline significance), indicating excessive practice.

Few studies present the variable “number of digital examinations” according to MoH and WHO recommendations. A study performed before the SN found excessive number of digital examinations, as no parturient was submitted to the recommended frequency of examinations (every four hours) ^(^ ^35^ ^)^ . After the SN, few studies have brought this data and, when they did it, they did not relate to the interval at which they were performed ^(^ ^27^ ^,^ ^49^ ^)^ .

It should be emphasized that the institution studied is a teaching hospital, which should not justify the repetitive performance of digital examinations. The WHO underlines that under no circumstances should women undergo frequent and repeated digital examinations by several professionals or students ^(^ ^1^ ^)^ .

Episiotomy also should not be routinely performed. Current evidence shows that its practice is not necessary and can even be harmful, leading to a number of complications, such as pain, dyspareunia, complications in subsequent deliveries, iatrogenic or spontaneous opening of the anal or rectal sphincter, unsatisfactory healing resulting in skin marks, asymmetry or excessive introitus narrowing, vaginal prolapse, recto-vaginal fistula, increased blood loss, edema, infection and dehiscence ^(^ ^50^ ^)^ . In addition to these complications, episiotomy is a violation of the sexual and reproductive rights of women because it is performed in a healthy body, without having a proven benefit and, in some cases, without the woman’s consent ^(^ ^51^ ^)^ and without previous local anesthesia. The WHO recommendation is not to prohibit episiotomy, but to restrict its use, which should not exceed a proportion of 10% in health facilities ^(^ ^1^ ^,^ ^52^ ^)^ .

A systematic review has concluded that the indication of episiotomy for the purpose of reducing perineal/vaginal trauma is not justified, nor is it sustained on the basis of current evidence ^(^ ^50^ ^)^ . A randomized clinical trial conducted in Recife/PE compared a protocol of selective episiotomy with a non-episiotomy protocol and showed that the non-episiotomy protocol seems to be safe for the woman and the newborn ^(^ ^53^ ^)^ .

Although the rate of episiotomy was reduced by 13.2% after the SN, most women included in this study (55.0%) continues to undergo this practice. National survey detected an episiotomy rate of 56.1% for women at usual risk and 48.6% for high risk women ^(^ ^5^ ^)^ , thus demonstrating - like the Kristeller’s maneuver - that the indication of episiotomy has no relation to maternal or fetal conditions. Studies evaluating the deliveries attended by midwife nurses demonstrate that it is possible to practice low rates of episiotomy and maintain quality of care, with a percentage of 15.4% ^(^ ^49^ ^)^ , 15.5% ^(^ ^11^ ^)^ and 25.7% ^(^ ^9^ ^)^ .

As evidenced throughout this discussion, being born in NDC, in a hospital with a collaborative model, in an institution certified by the BFHI, or having a midwife-assisted delivery increases the chances of women and their newborns have access to good practices and reduces the chances of harmful and unnecessary interventions.

Some limitations of this study were due to incomplete or non-existent records, such as the impossibility of the evaluation of the partograph and of the measurement of skin-to-skin contact time.

## Conclusion

The implementation of evidence-based delivery and birth practices is characterized as a highly effective strategy to improve maternal and neonatal outcomes and is also an action to ensure the sexual and reproductive rights of women. Considering its impact on obstetric care, evidence-based practices have been systematically recommended since 1996 by the World Health Organization. In Brazil, these practices were first recommended in 2001 through the manual “Childbirth, miscarriage and puerperium: humanized care to the woman”, but they were only officially incorporated into SUS in 2011 through the Stork Network. Thus, the monitoring thereof is an important strategy to qualify childbirth care for institutions.

Even before the implementation of the Stork Network, the maternity ward studied had already incorporated evidence-based practices under WHO recommendations. It showed good results for the good practices (Category A) evaluated, except for the variable encouragement to breastfeeding soon after birth, which presented an increase, but with an index below that expected, and for the variable freedom of position and movement, which presented a reduction, even though it is a cost-free practice and has a direct impact on the evolution of labor. None of the harmful practices (Category B) assessed were discontinued, even four years after the Stork Network, with a significant increase in venoclysis and the almost universal maintenance of the lithotomy position in the expulsive period, although without significant difference.

On the practices originally classified as without sufficient evidence (Category C), we highlight the significant increase of Kristeller’s maneuver. There is nowadays considerable evidence about this practice and current research considers Kristeller’s maneuver to be obstetric violence, requiring its use to be urgently revised. In the analysis of the inappropriate practices frequently used (Category D), the proportion of the number of digital examinations above the recommended increased and the episiotomy rate decreased; however, most of the women continued to undergo this latter practice and, in some cases, without previous local anesthesia, denoting the continuity of its improper and routine use. Despite this, there was an increase in the supply of analgesia, which, from a perspective, represents the appreciation of women’s right to obtain pharmacological resources for pain relief and for another perspective represents the medicalization of the body and a care routine little committed to ensuring a less invasive and with less risk delivery care.

These findings reveal the maintenance, over four years of the Stork Network, of both good and harmful and inappropriate practices, indicating that officially advocating in a government policy the use of evidence-based practices is effective to reinforce and ensure their continuity, but it is insufficient to reverse, alone, the pattern of unnecessary and harmful interventions. A consolidated path worldwide is the inclusion of midwife nurses in the care and clinical decision making during the labor process, providing for the organization of work in a shared configuration, under the logic of the collaborative model. This model of care has the potential of implementing evidence-based practices and reversing unfavorable rates of maternal and neonatal morbidity and mortality through the appropriate use of technologies and the reduction of unnecessary interventions.
